# A Conserved Role for Syndecan Family Members in the Regulation of Whole-Body Energy Metabolism

**DOI:** 10.1371/journal.pone.0011286

**Published:** 2010-06-23

**Authors:** Maria De Luca, Yann C. Klimentidis, Krista Casazza, Michelle Moses Chambers, Ruth Cho, Susan T. Harbison, Patricia Jumbo-Lucioni, Shaoyan Zhang, Jeff Leips, Jose R. Fernandez

**Affiliations:** 1 Department of Nutrition Sciences, University of Alabama at Birmingham, Birmingham, Alabama, United States of America; 2 Nutrition and Obesity Research Center, University of Alabama at Birmingham, Birmingham, Alabama, United States of America; 3 Diabetes Research Training Center, University of Alabama at Birmingham, Birmingham, Alabama, United States of America; 4 Section on Statistical Genetics, Department of Biostatistics, University of Alabama at Birmingham, Birmingham, Alabama, United States of America; 5 Department of Biological Sciences, University of Maryland Baltimore County, Baltimore, Maryland, United States of America; 6 Department of Genetics, North Carolina State University, Raleigh, North Carolina, United States of America; University of Texas MD Anderson Cancer Center, United States of America

## Abstract

Syndecans are a family of type-I transmembrane proteins that are involved in cell-matrix adhesion, migration, neuronal development, and inflammation. Previous quantitative genetic studies pinpointed Drosophila *Syndecan* (*dSdc*) as a positional candidate gene affecting variation in fat storage between two *Drosophila melanogaster* strains. Here, we first used quantitative complementation tests with *dSdc* mutants to confirm that natural variation in this gene affects variability in Drosophila fat storage. Next, we examined the effects of a viable *dSdc* mutant on Drosophila whole-body energy metabolism and associated traits. We observed that young flies homozygous for the *dSdc* mutation had reduced fat storage and slept longer than homozygous wild-type flies. They also displayed significantly reduced metabolic rate, lower expression of *spargel* (the Drosophila homologue of PGC-1), and reduced mitochondrial respiration. Compared to control flies, *dSdc* mutants had lower expression of brain insulin-like peptides, were less fecund, more sensitive to starvation, and had reduced life span. Finally, we tested for association between single nucleotide polymorphisms (SNPs) in the human *SDC4* gene and variation in body composition, metabolism, glucose homeostasis, and sleep traits in a cohort of healthy early pubertal children. We found that SNP rs4599 was significantly associated with resting energy expenditure (*P* = 0.001 after Bonferroni correction) and nominally associated with fasting glucose levels (*P* = 0.01) and sleep duration (*P* = 0.044). On average, children homozygous for the minor allele had lower levels of glucose, higher resting energy expenditure, and slept shorter than children homozygous for the common allele. We also observed that SNP rs1981429 was nominally associated with lean tissue mass (*P* = 0.035) and intra-abdominal fat (*P* = 0.049), and SNP rs2267871 with insulin sensitivity (*P* = 0.037). Collectively, our results in Drosophila and humans argue that syndecan family members play a key role in the regulation of body metabolism.

## Introduction

Obesity is a condition characterized by an excess of adipose tissue that adversely affects human health [1]. The clinical problem of excessive adipose tissue resides in its strong association with a number of chronic diseases, such as insulin resistance, type 2 diabetes mellitus (T2DM), coronary artery disease and stroke [1]. In 2007–2008, 33.8% of the adults in the United States were obese [2]. Another worrisome observation is the increase in percent of obese children and adolescents [3]. Obese children are more likely to become obese adults [4], and thus we may see profound public health consequences as a result of the appearance of associated co-morbidities in adulthood.

The role of genes in human obesity and related phenotypes is well established [5], [6]. The fruit fly *D. melanogaster* has emerged in recent years as a powerful model organism for studying the genetics of fat storage and obesity [7]–[9]. Previously, we used a recombinant mapping approach to identify chromosomal regions (quantitative trait loci or QTL) that contribute to variation in triacylglycerol (TAG) storage using two unrelated strains of *D. melanogaster*, *Oregon R* (*Ore*) and *2b* [10]. Subsequent fine mapping of these QTL regions identified several candidate genes that contribute to variation in TAG storage, many of which have been verified to be important for TAG storage in both Drosophila and humans [7]. Fine-mapping localized one QTL affecting TAG to the 57E1;57E3 cytogenetic region on chromosome 2 [10]. Only five genes lie within this region, including the *dSdc* gene, which encodes a member of the syndecan gene family [11].

Syndecans are type-I transmembrane proteins that are present on the surface of all adherent cells [12]. While Drosophila appears to have only one syndecan protein, mammals have four syndecan proteins encoded by four separate genes. Three of them, *SDC1*, *SDC2*, and *SDC3*, are expressed in a tissue-specific manner, whereas the fourth, *SDC4*, is expressed in a variety of cell types [12]. All syndecan proteins are characterized by a core protein composed of an extracellular domain (ectodomain) followed by a single hydrophobic membrane-spanning domain and a short intracellular domain. The ectodomain contains attachment sites for heparan sulphate polysaccharide chains that mediate interactions with extracellular matrix (ECM) components [13], heparin-sulfate growth factors [14], cell adhesion molecules [15], lipases [16], chemokines, cytokines and their receptors [17], and pathogens [18]. As a result, syndecans function as co-receptors modulating signal transduction pathways initiated by growth factors and are involved in cell proliferation, adhesion and migration, lipid metabolism, and inflammation [12]. Via their intracellular domains, syndecans also interact with cytoplasmic proteins that control focal adhesion, cell spreading, and actin cytoskeletal organization [12], hence playing a direct role in transducing signals from the ECM to the cytoplasm. Previous studies confirmed the functional conservation of syndecan in Drosophila [19]. Furthermore, Drosophila syndecan has been reported to participate in normal axon guidance and neuronal development via regulation of the Slit/Roundabout signaling [20].

Based on these observations, the results of our genetic mapping experiment, and the similarity in structural characteristics between Drosophila and mammalian syndecans, we reasoned that *dSdc* was a candidate gene contributing to variation in TAG storage between *Ore* and *2b*. To investigate this hypothesis, we performed quantitative complementation tests by crossing mutants of *dSdc* with flies of the *Ore* and *2b* genotypes and found that allelic differences at *dSdc* produced differences in TAG storage between these two strains. We next examined the functional role played by *dSdc* in fat storage by testing whether flies homozygous for a hypomorphic mutation of *dSdc* (*dSdc^BG^* ^02774^) and flies homozygous for the corresponding wild-type allele differed in whole-body energy metabolism and associated traits in Drosophila. Collectively, our results in Drosophila showed that dSdc plays an important role in the regulation of body composition and metabolism in young flies.

In humans, the *SDC4* gene includes 5 exons spanning 19.7 kb on chromosome 20q12 [21]. A number of whole-genome linkage studies have linked the chromosomal region 20q12-13 to T2DM and obesity [22]–[25]. Based on this observation and our results in Drosophila, we carried out an explorative study to examine the association between genetic variants in the human *SDC4* gene and variation in traits associated with body composition, energy metabolism, and sleep duration in a cohort of 252 healthy early pubertal children. Although small in size, this human cohort was chosen for two main reasons. First, the phenotypic data available for each subject included robust measurements of body composition and glucose-insulin dynamics. Second, the cohort was characterized by genetic admixture, which allowed us to adjust for ancestry within ethnic groups and therefore limit false-positive results [26]. Consistent with our data in flies, association tests using *SDC4* genotypes as independent variables revealed an impact of *SDC4* variation on resting energy expenditure (REE), insulin dynamics, and sleep duration in our cohort of young children. These results motivate future genetic studies in independent human populations to verify the effects of this gene.

## Results

### Complementation tests to *dSdc* mutations

**Figure 1:**
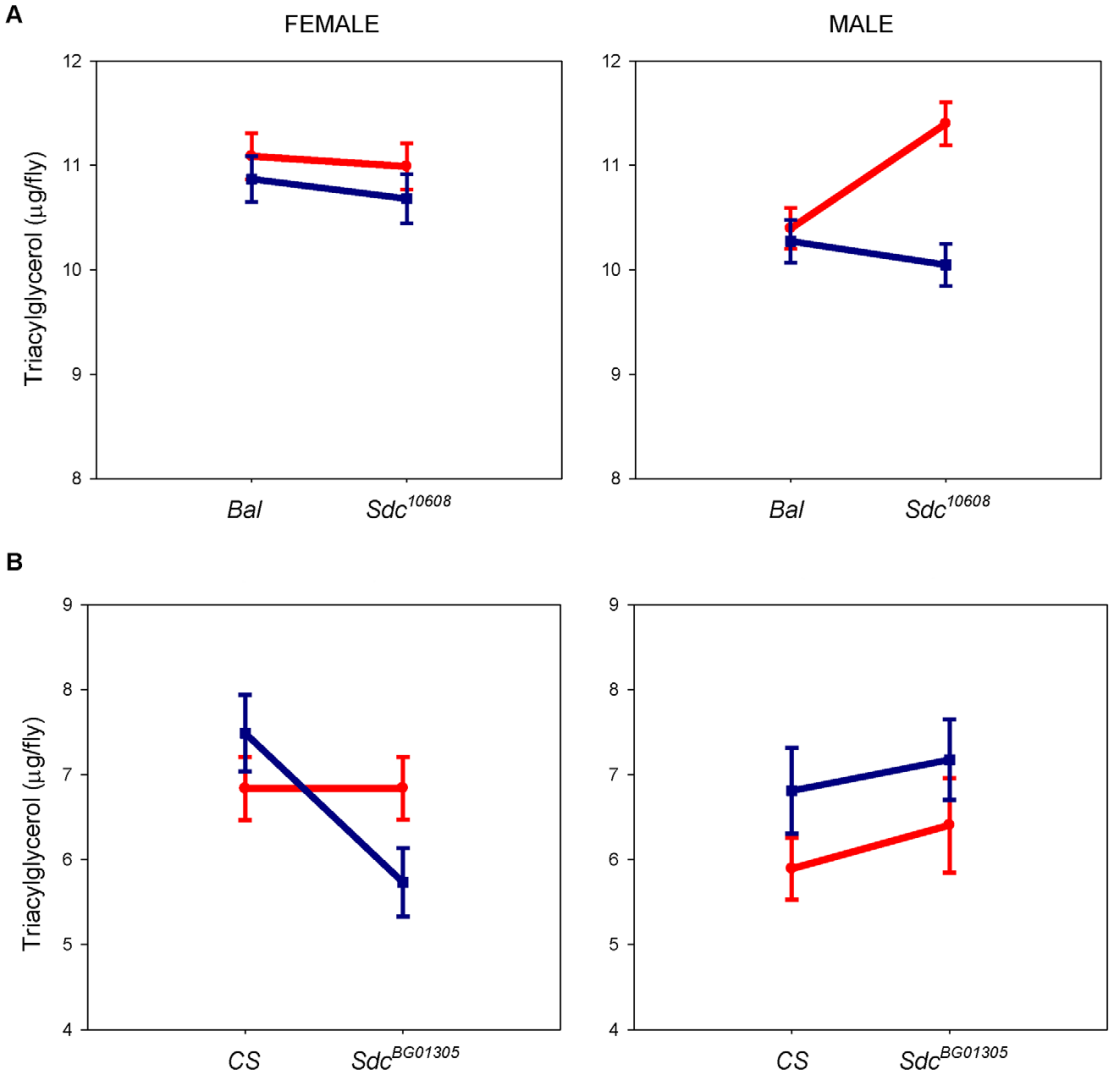
Variation in *dSdc* affects variability in *Drosophila* fat storage. (A and B) Quantitative complementation tests with two mutations of *dSdc*. TAG levels are assessed for four genotypes: *M*/2b, *Bal or CS*/2b, *M*/Ore, andz *Bal or CS*/Ore, where *M* denotes the *dSdc* mutation, *Bal* is the balancer chromosome, and *CS* is the *Canton S* strain. Values shown are the TAG least-squared means within genotype classes for *n* = 10 independent replicates. Details on the analysis are provided in the text. *Ore* and *2b* heterozygote flies are color-coded red and dark blue, respectively. (A) In females, there is no evidence of quantitative failure of the *Sdc^10608^* mutation to complement the *Ore* and *2b* alleles because the relative difference in the average trait values between *Ore* and *2b* over *Sdc^10608^* mutation and the *Bal* is the same. In males, however, the difference in the average trait values between *Ore* and *2b* alleles over the *Sdc^10608^* mutation is greater than the difference in the average trait value between *Ore* and *2b* alleles over the *Bal* (*P* value for Line x Genotype is 0.003), which indicates failure of the *Sdc^10608^* to complement the *Ore* and *2b* alleles. (B) Although there is no evidence of quantitative failure to complement in males, greater difference in female *Ore* and *2b* lines when over *Sdc^BG01305^* mutation compared to female *Ore* and *2b* over the *CS* strain indicates failure of the *Sdc^10608^* to complement the *Ore* and *2b* alleles (*P* value for Line x Genotype is 0.033). Collectively, these results indicate an interaction between the mutated allele (*Sdc^10608^* or *Sdc^BG01305^*) and the TAG QTL of *Ore* and *2b*.

To investigate the relationship between *dSdc* and TAG storage, we first used complementation tests with two mutations of *dSdc* (*Sdc^10608^* and *Sdc^BG01305^*). *Sdc^10608^* failed to complement the TAG phenotype of *Ore* and *2b* QTL in males (Fig. 1A) and *Sdc^BG01305^* failed to complement the TAG phenotype of *Ore* and *2b* QTL in females (Fig. 1B). The allelic effects of variation at this locus were consistent for both *dSdc* mutants, as flies with the *Ore* allele had higher levels of TAG than flies with the *2b* allele. Thus, these results confirmed *dSdc* as a candidate gene affecting variation in TAG storage between these two strains of *D. melanogaster*.

### Effect of *Sdc^BG02774^* mutation on body weight mutation on body weight, total protein content, and metabolite storage

**Figure 2:**
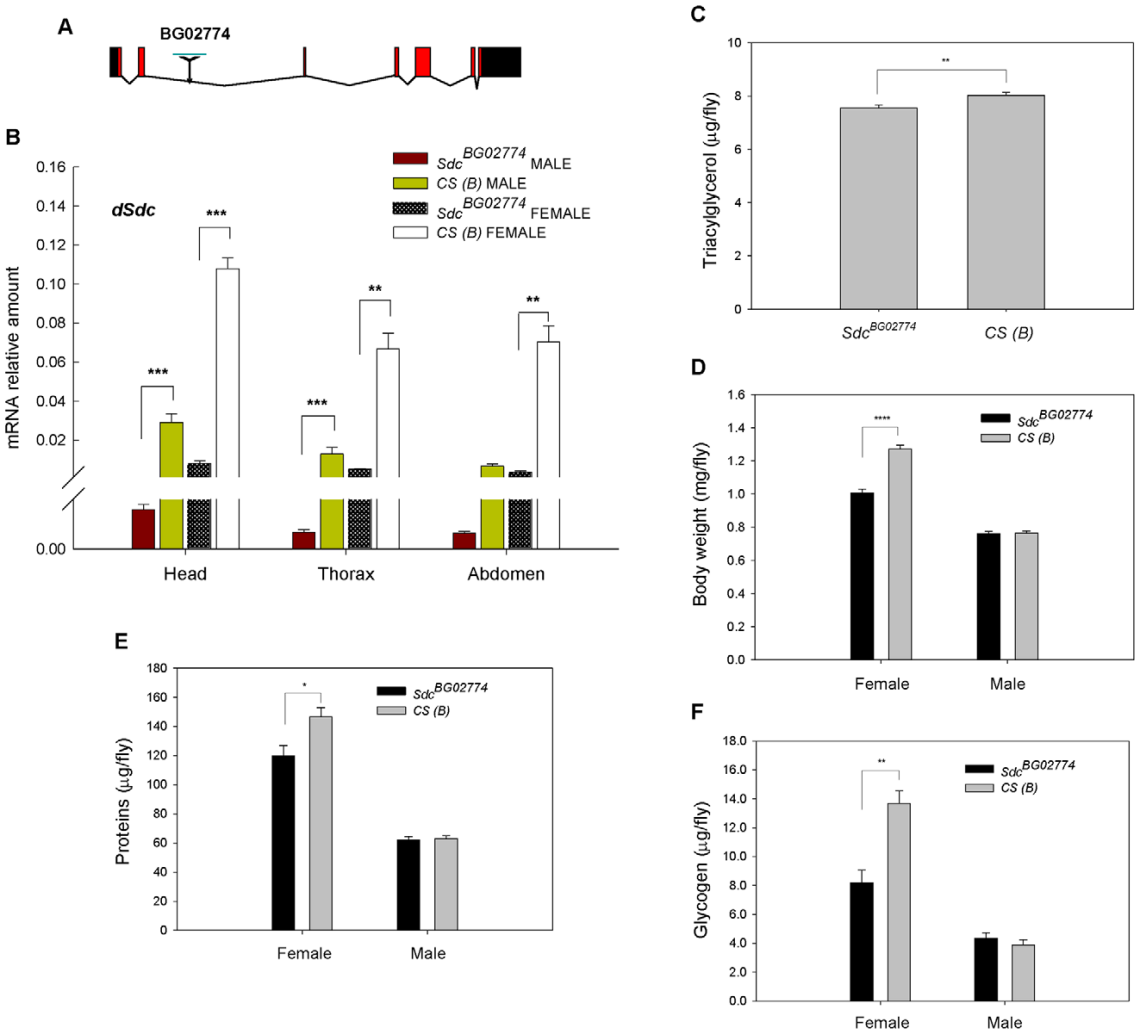
Effect of *Sdc^BG0277^* ^**4**^ mutation on body weight, total protein content, and metabolite storage. (A) Schematic representation of *dSdc* gene region on the second chromosome at cytological position 57E1-57E6 (NCBI accession no. AE013599.4). Black and red boxes represent untranslated regions and exons, respectively. The location of the *p[GT1]* insertion site that creates the *Sdc^BG0277^* ^4^ mutation is indicated with an arrowhead. (B) *P*-element insertion in the *dSdc* gene leads to significantly reduced *dSdc* transcript abundance. Messenger RNA levels analyzed by RT-qPCR (*n* = 6) on cDNA using primers that encompass a common region of alternative transcripts. Levels of *dSdc* mRNA were normalized to Drosophila *ribosomal protein49* (*rp49*) mRNA levels. (C) *Sdc^BG0277^* ^4^ mutation affects TAG levels. Because no differences were observed between male and female flies in TAG storage, we pooled male and female data for the analysis. Values represent least-square means from *n* = 20 independent replicates of homozygous *Sdc^BG0277^* ^4^ and *CS* (*B*) flies. (D–F) *Sdc^BG0277^* ^4^ mutation affects body weight, total protein content, and glycogen levels in females. Each value represents the mean body weight (panel D) and least-square means for protein (panel E) and glycogen (panel F) levels from *n* = 10 independent replicates. In all panels, error bars represent SEM. * *P*≤0.05, ** *P*≤0.01, *** *P*≤0.001, **** *P*≤0.0001 compared to control.

To examine the functional role played by *dSdc* in fat storage, we used a viable mutant allele of the gene, *Sdc^BG02774^*. *Sdc^BG02774^* is a mutant generated in the *w^1118^*;*Canton S* (*B*) [*CS* (*B*)] strain by the insertion of a *p[GT1]*-element in the second intron of *dSdc* (Fig. 2A) [27]. We examined the effects of this *P*-element insertion on *dSdc* transcription in adult flies by performing Real Time-quantitative PCR (RT-qPCR) experiments using RNA isolated from three body parts (head, thorax, and abdomen). We found that the overall expression of *dSdc* is significantly reduced in the three body parts of *Sdc^BG02774^* flies (Fig. 2B).

Next, we assessed the effect of the *Sdc^BG02774^* mutation on body weight and whole-body TAG, protein, and glycogen contents. After adjusting for body weight, we found a small but significant reduction in TAG storage in homozygous *Sdc^BG02774^* flies compared to controls (Fig. 2C). On average, TAG levels of *Sdc^BG02774^* flies were 6% lower than that of controls. However, sex-specific effects were observed for the other traits. Compared to controls, females homozygous for the *Sdc^BG02774^* mutation had significantly reduced body weight (21%) and body weight-adjusted protein (18%) and glycogen (40%) contents, while male *Sdc^BG02774^* did not differ from the control strain (Fig. 2D–F).

### Homozygous *Sdc^BG02774^* flies have lower expression levels of *dilps*, are less fertile, and have reduced metabolic activity than controls

**Figure 3:**
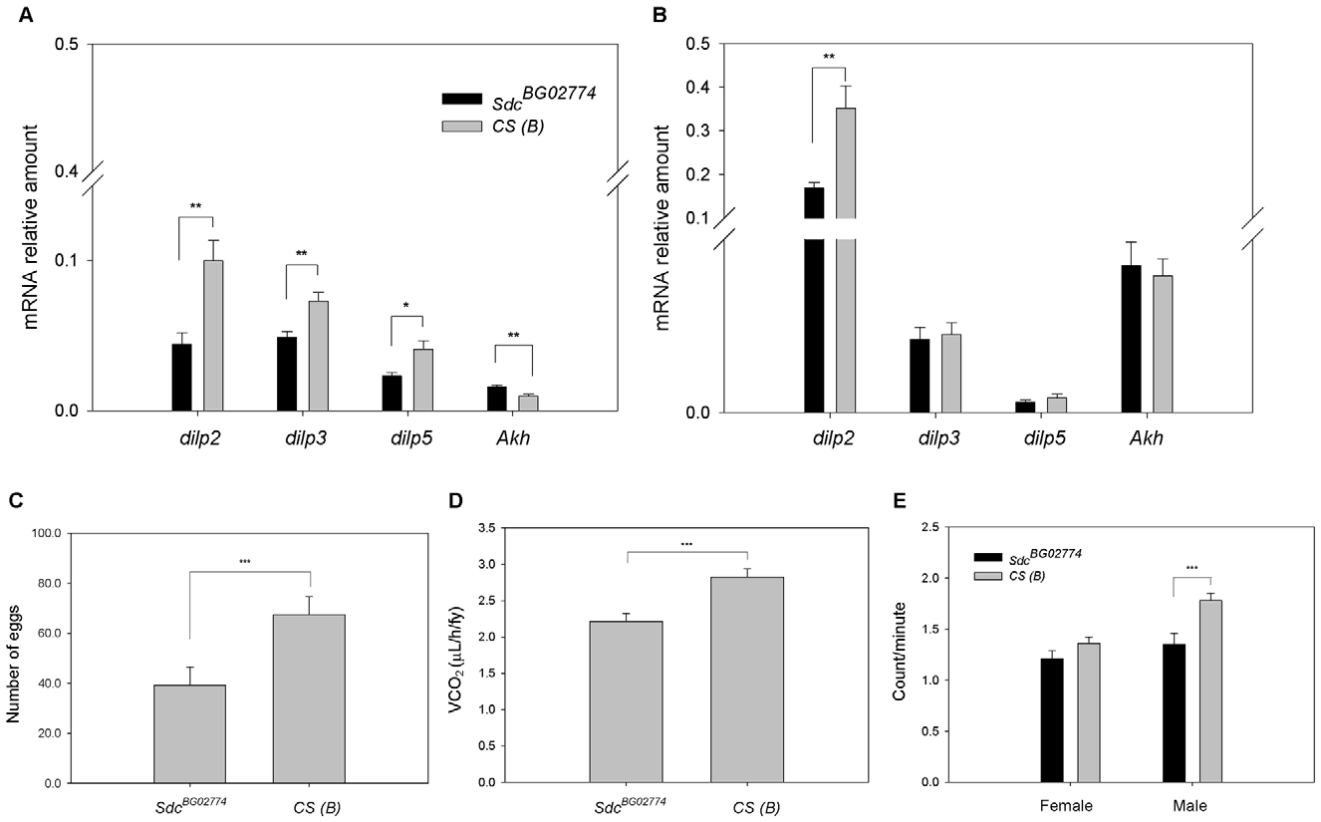
Homozygous *Sdc^BG0277^* ^**4**^ flies have lower expression levels of *dilps*, fecundity, and metabolic activity. (A–B) Gene expression levels were measured by RT-qPCR using mRNA extracted from heads (*dilps*) and whole-body (*Akh*) of female (panel A) and male (panel B) flies (*n* = 6). All genes were normalized to *rp49*. (C) Values represent the average number of eggs laid by female flies over a five day period per *n* = 10 independent replicates. (D) CO_2_ production measured by indirect calorimetry. No differences were observed between male and female flies and the values represent VCO_2_ least-square means of *n* = 20 independent replicates. (E) Waking activity was measured counting the number of times a given fly crosses an infrared beam during a one-minute interval. Values represent the average number of activity counts per waking minute of two independent replicates of 16 flies. In all panels, error bars represent SEM. *P≤0.05, ** *P*≤0.01, *** *P*≤0.001 compared to control.

In insects, the insulin/insulin-like growth factor (IGF) signaling controls organ growth and final body size [28] as well as metabolism in adult flies [29], [30]. The Drosophila genome contains seven Drosophila insulin-like peptide (*dilp*) genes. Several of the dilps (*dilp1-5*) are expressed at high levels in insulin producing cells (IPC) of the *pars intercerebralis* in the brain of both larvae [31] and adult flies [29]. To investigate whether the *Sdc^BG02774^* had a defect in the production of DILPs, we measured mRNA levels of *dilp2, dilp3*, and *dilp5*, which are expressed in the IPC of adult flies [29], in *dSdc* mutants and controls. We also investigated the expression level of the gene that encodes the adipokinetic hormone (AKH), a putative glucagon homolog. Like pancreatic insulin and glucagon-producing cells in mammals, cross-regulatory interactions exist between DILP- and AKH-producing cells in Drosophila [32] and expression levels of *Akh* were previously shown to be increased in IPC-deficient larvae and adults [33]. In females, we observed a 55%, 33% and 43% reduction in expression of *dilp2*, *dilp3* and *dilp5*, respectively, and a 38% increase in *Akh* expression in *Sdc^BG0277^* ^4^ flies compared with controls (Fig. 3A). In males, we found no difference between mutants and controls for *dilp3*, *dilp5*, and *Akh*, but the *Sdc^BG0277^* ^4^ flies showed a 52% reduction in expression of *dilp2* (Fig. 3B).

Recently, Zhang and collaborators [30] have shown that flies homozygous for a deletion of *dilp1-5* were poorly fertile and had reduced metabolic activity. A significant reduction in egg-production was also observed by Gronke *et al*. [34] in *dilp2*-*3,5* mutant females. Therefore, we tested the impact of the *dSdc* mutation on these phenotypes. First, we measured fecundity by counting the total number of eggs laid over a five day period. As expected, we found that *dSdc^BG02774^* females laid significantly less eggs (42% less) than control flies (Fig. 3C). Next, we examined metabolic rate by measuring carbon dioxide (CO_2_) production. Because no differences were observed between male and female flies in CO_2_ production, we pooled male and female data for statistical analysis. We observed that *dSdc* mutants displayed a 22% reduction in metabolic rate as compared to controls (Fig. 3D). Finally, we monitored waking activity, the number of times flies crossed an infrared beam per minute spent awake [35]. We did not observe a difference in waking activity in *Sdc^BG02774^* female flies; however, *Sdc^BG02774^* male flies showed a 24% reduction in waking activity compared to controls (Fig. 3E).

### Homozygous *Sdc^BG02774^* flies have lower transcript levels of *Thor* and *spargel* and reduced mitochondrial function compared to controls

**Figure 4:**
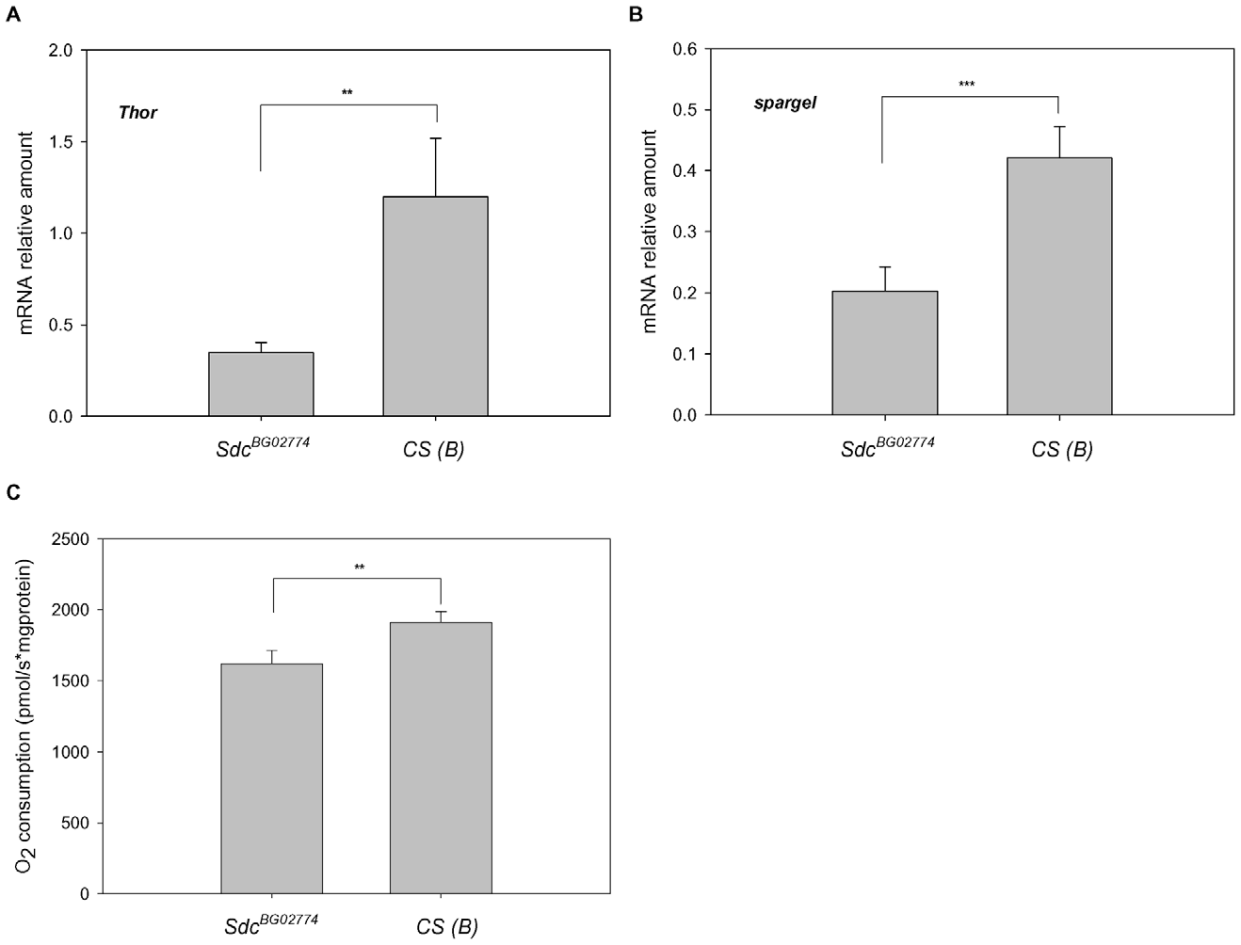
Homozygous *Sdc^BG02774^* flies have lower expression levels of *spargel* and reduced mitochondrial function. (A–B) Despite reduced levels of DILPs, homozygous *Sdc^BG02774^* flies do not show increased levels of *Thor*, but display lower levels of *spargel* mRNA than controls. Gene expression levels were measured by RT-qPCR using mRNA extracted from whole body of *Sdc^BG02774^* and *CS (B)* flies. Transcript levels of *Thor* and *spargel* were normalized to *rp49*. Values represent average of 17 (panel A) and 12 (panel B) independent replicates. (C) O_2_ consumption by whole-fly isolated mitochondria was measured in the presence of saturating amounts of NAD^+^-linked respiratory substrates (pyruvate *plus* proline) and ADP (state 3 respiration rate). Values represent average of 20 independent replicates. In all panels, pooled male and female data was used for the analysis. Error bars represent SEM. ** *P*≤0.01, *** *P*≤0.001 compared to control.

Reduced expression of *dilps* should lead to systemic reduction of the insulin signaling pathway. To assess whether the insulin signaling is indeed reduced in the *dSdc* mutants, we first investigated the insulin-dependent transcriptional response induced by the Drosophila forkhead transcription factor (dFoxO). Reduced insulin receptor/PI3K/Akt signaling in Drosophila induces nuclear accumulation of the dFoxO, which in turn activates the transcription of target genes, such as the translational regulator *Thor*, the Drosophila homolog of mammalian *EIF4EBP1* [36]. We measured *Thor* mRNA levels in *dSdc* mutants and controls and found no differences between male and female flies; therefore we pooled the data for the analysis. Interestingly, in contrast to our prediction, we observed that *Thor* expression was significantly reduced in *dSdc* mutants (71%) compared to control flies (Fig. 4A), despite the fact that mutants exhibited reduced levels of DILPs (Fig. 3A,B).

Peroxisome-proliferator-activated receptor-gamma co-activator-1 (PGC-1) family members play a pivotal role in the control of energy homeostasis in mammals [37]–[39]. Recently, Tiefenböck *et al.* [40] have shown that the Drosophila homologue of PGC-1, Spargel, is a critical downstream component for insulin signaling. The authors reported that Spargel acts in parallel to dFOXO and mediates mitochondrial respiration in response to insulin signaling [40]. To determine whether reduced levels of *dilps* in *Sdc^BG02774^* flies might lead to a decrease in the expression of *spargel* gene, we compared mRNA levels of *spargel* in *Sdc^BG02774^* and *CS (B)* flies. Once again, we did not observe differences in transcript levels between male and females and therefore we pooled the data for the statistical analysis. We found that mRNA levels of *spargel* were reduced 52% in the mutant flies relative to controls (Fig. 4B).

Finally, we analyzed respiration rates in mitochondria isolated from mutant and control flies using NAD^+^-linked respiratory substrates (pyruvate *plus* proline) that deliver electrons into complex I of the mitochondrial electron transport chain. In the analysis averaged across sexes, we observed that the ADP-dependent state 3 respiration rate (a measure of oxidative phosphorylation capacity) was reduced by approximately 15% in *Sdc^BG02774^* compared with the controls (Fig. 4C).

### Homozygous *Sdc^BG02774^* flies have reduced survival and are more sensitive to starvation than controls

**Figure 5:**
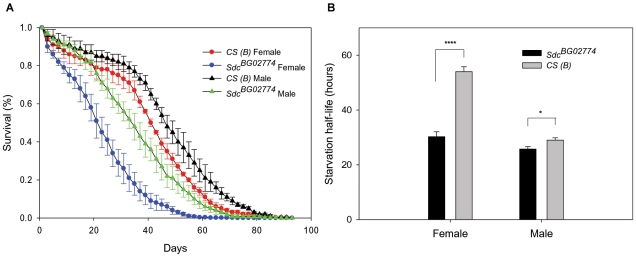
Homozygous *Sdc^BG02774^* flies have reduced lifespan and are more sensitive to starvation. (A) Life span assays were carried out using population cages. We used three replicates of each sex and genotype combination with initial population sizes ranging from 183 to 295 individuals. Survival was monitored every other day until all flies in a population cage had died. Mutants of both sexes had significantly reduced survival compared with controls (Females and Males: *P*<0.0001). (B) Values represent the average starvation half-life of *n* = 5 independent replicates. In both panels, error bars represent SEM. *P≤0.05, **** *P*≤0.0001 compared to control.

Ablation of the IPCs in the *pars intercerebralis* of the final instar larval brain results in adult flies with reduced fecundity, increased resistance to starvation, and extended life span [29]. An increase in survival has also been observed in *dilp2* mutant flies [34] and in flies with reduced expression levels of *dilp2* as a result of genetic manipulation of dFoxO [41], c-*Jun* N-Terminal Kinase [42], or p53 [43]. Thus, we asked whether the reduced *dilps* transcript levels observed in the *dSdc* mutant flies would affect survival under normal feeding and water-only starvation conditions. We found significant differences in survival between *Sdc^BG02774^* mutants and controls. However, contrary to our prediction, median life span of female and male *Sdc^BG02774^* flies was decreased by 46% and 28%, respectively, under normal feeding conditions (Fig. 5A). Female and male mutant flies were also less resistant to starvation than controls by 45% and 11%, respectively (Fig. 5B).

### Homozygous *Sdc^BG0277^* ^4^ flies sleep longer than controls

**Figure 6:**
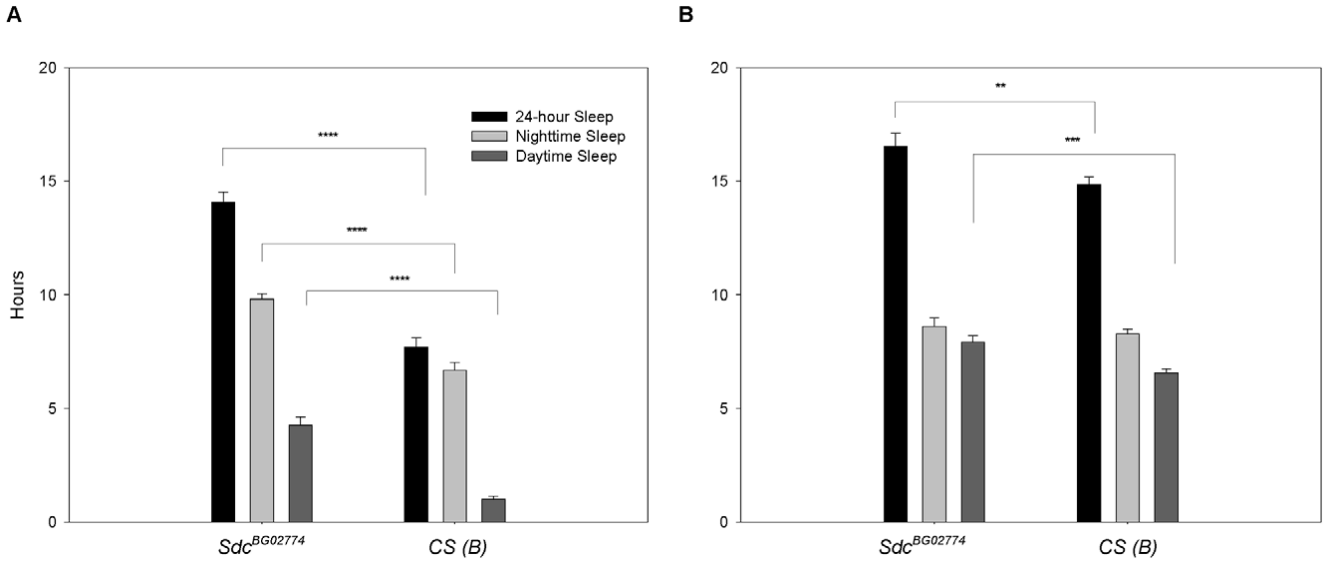
Homozygous *Sdc^BG02774^* flies sleep longer. (A–B) Sleep parameters were measured counting the number of times a given fly crosses an infrared beam during a one-minute interval. Sleep was defined as any period 5 minutes or longer without an activity count. Values represent average hours of sleep of two independent replicates of 16 female (panel A) and male (panel B) flies. In both panels, error bars represent SEM. ** *P*≤0.01, *** *P*≤0.001, **** *P*≤0.0001 compared to control.

Previously, Harbison and collaborators [44] reported a significant correlation between two genetic variants in the *dSdc* gene and daytime sleep in 40 wild-derived Drosophila lines, suggesting that *dSdc* may also be involved in sleep duration. To test this hypothesis, we assessed the sleep-wake cycle in *dSdc^BG^* ^02774^ and *CS (B)* flies over the course of a 24 h day. As expected, sleep was increased by 32% during nighttime and by 76% during daytime in *dSdc^BG^* ^02774^ female flies compared to controls (Fig. 6A). A 17% increase in daytime sleep was also observed in mutant males (Fig. 6B).

### Variation in human *SDC4* is associated with energy metabolism, body composition, and sleep

**Figure 7:**
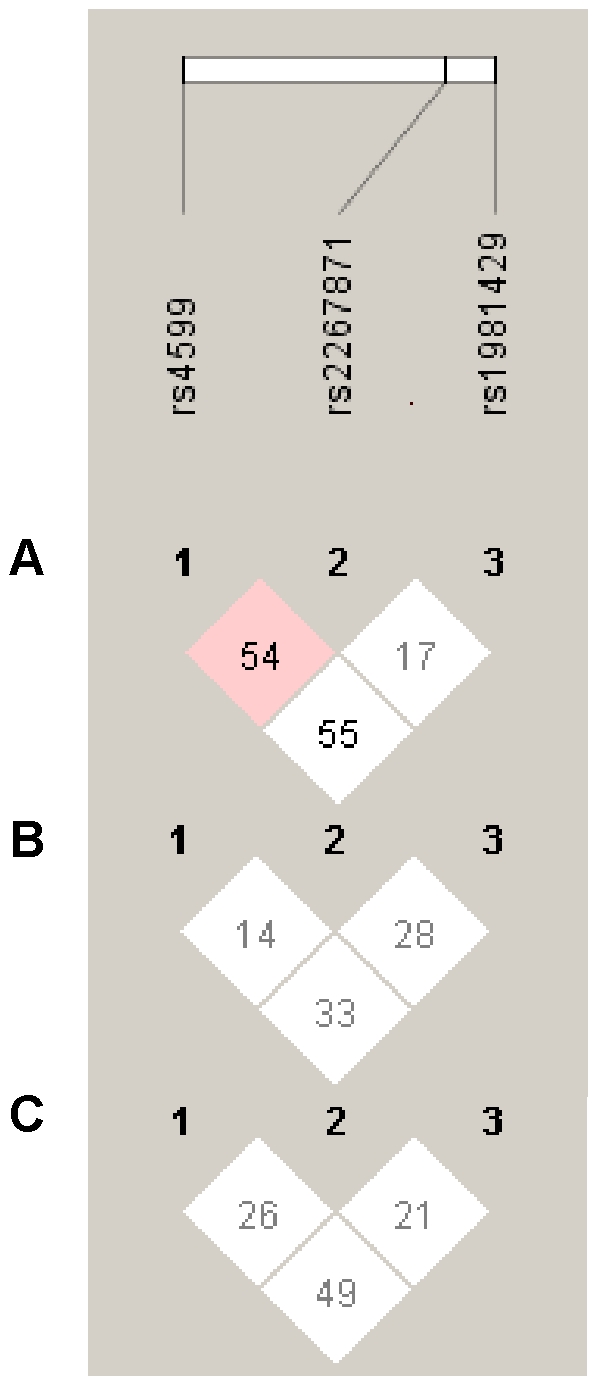
Pair-wise linkage disequilibrium (D') pattern between three *SDC4* SNPs in the human cohort. Plots generated using Haploview v3.2 [89]. The color code on the plots follows the Haploview Standard Color D'/LOD Scheme: shades of pink/red (D'<1; LOD ≥2); white (D'<1; LOD<2). The numbers represent D' values expressed as a percentage. (A) European Americans. (B) African Americans. (C) Hispanic Americans.

We conducted a population-based association study on 252 European American (EA), African American (AA) and Hispanic American (HA) children. Anthropometric and metabolic characteristics of the study subjects are shown in Table 1. We selected three haplotype-tagging SNPs (htSNPs) that map within the *SDC4* gene from the International Haplotype Map (HapMap) Project (http://www.hapmap.org): rs1981429 (T/G) and rs2267871 (T/A), which map in the intron 2, and rs4599 (C/T) in the 3′UTR region. All genotype groups were in Hardy-Weinberg equilibrium (Table 2). The Minor Allele Frequencies ranged from 0.11 to 0.49 (Table 2). There was no difference in genotype frequencies between ethnic groups for rs2267871. However, there was a significant difference in genotype frequencies between ethnic groups for rs1981429 (*P*<0.0001) and rs4599 (*P* = 0.0002), which is consistent with genotypic data reported by the HapMap Project. Low to moderate pair-wise linkage disequilibrium estimates were observed among the SNPs in the three ethnic groups (Fig. 7).

**Table 1:** Characteristics of human subjects.

	All (n = 252)	Hispanic Americans (n = 58)	European Americans (n = 108)	African Americans (n = 86)
Sex (%male)	51.6	46.6	52.8	53.5
Age (yrs)	9.7±1.6	9.4±1.5	9.7±1.7	9.7±1.5
BMI *	18.5±2.9	19.4±2.7	18.0±2.6	18.6±3.3
Weight (kg)	36.9±9.1	37.1±8.5	35.9±8.8	37.9±10.0
Total Lean Mass (kg) **	26.0±5.2	24.7±4.7	8.4±5.1	27.5±5.5
Total Fat Mass (kg) *	8.8±5.5	10.4±5.1	8.4±5.1	8.17±6.05
IAAT (cm^2^)**	33.6±22.7	42.1±24.7	34.9 ±22.8	26.7±18.7
SI [×10^−4^ min^−1^/(µU/mL)]***	5.8±3.6	5.4±3.8	7.0 ±3.9	4.55±2.6
Fasting Glucose (mg/dL)***	97.0±6.4	99.6±6.1	97.5±6.2	94.5±5.9
REE (kcal/day)	1195±243	1191±292	1194 ±242	1199±204
Hours of sleep/night ***	8.9±1.1	8.85±1.22	9.3±0.8	8.53±1.15

Values are means ± SD. BMI: body mass index. IAAT: Intra-abdominal adipose tissue. SI: Insulin sensitivity index. REE: Resting energy expenditure. *P* values for difference between ethnic groups are obtained using ANOVA.

* *p*<0.05,

** *p*<0.01 and

*** *p*<0.001.

**Table 2:** SNP marker information at the human *SDC4* locus in the study cohort.

dbSNP rs #	Alleles	Genomic Position*	Heterozygosity			HWE *P*-value			MAF			
			HA	EA	AA	HA	EA	AA	HA	EA	AA	
rs1981429	T/G	43409107	0.43	0.50	0.47	0.74	1.00	0.66	0.27	0.49	0.36	
rs2267871	T/A	43405784	0.31	0.28	0.22	1.00	0.34	0.59	0.19	0.19	0.11	
rs4599	C/T	43387821	0.29	0.31	0.53	0.26	1.00	0.17	0.23	0.18	0.36	

*Position in [91]. HWE: Hardy-Weinberg Equilibrium. MAF: Minor Allele Frequency.

We next examined whether the SNPs were independently associated with each trait. To account for the confounding effects of population stratification, we used estimates of genetic admixture as a covariate in statistical models (see Materials and Methods below). Age, gender, Tanner status, and appropriate potential confounding variables were also included in the analysis as covariates (see Table 3 for details). We found that SNP rs4599 was significantly associated with REE (the conservative Bonferroni corrected significance threshold is *P* = 0.0038) and nominally associated with fasting glucose levels and sleep duration (Table 3). On average, children homozygous for the C allele had 4% lower levels of glucose, 8% higher REE, and slept 7% less than children homozygous for the T allele. The association with sleep duration was also observed with SNP rs2267871 (Table 3). In this case, the *P* value was smaller which suggests that SNP rs2267871 is the site with the largest association with the true causal polymorphism.

**Table 3:** Least square means (95% lower and upper confidence intervals) for body composition, metabolic, and sleep traits of study subjects stratified according to *SDC4* polymorphisms.

				P*
*rs4599*	C/C	T/C	T/T	
n	16	96	140	
BMI	18.94 (17.66–20.30)	18.57 (18.03–19.13)	18.16 (17.72–18.61)	0.126^a^, 0.156**^d^**, 0.347^r^
Weight (kg)	37.24 (34.8–39.84)	36.26 (35.25–37.3)	35.59 (34.47–36.43)	0.164, 0.228, 0.305
TFM (kg)	7.74 (6.09–9.83)	7.63 (6.92–8.42)	7.32 (6.75–7.94)	0.534, 0.526, 0.788
LTM (kg)	26.22 (25.14–27.34)	25.74 (25.3–26.18)	25.38 (25.02–25.75)	0.087, 0.131, 0.232
IAAT (cm^2^)	30.78 (24.97–37.94)	27.85 (25.49–30.42)	28.85 (26.74–31.14)	0.919, 0.778, 0.441
SI [×10^−4^ min^−1^/(µU/mL)]	4.65 (3.65–5.91)	5.28 (4.80–5.80)	4.70 (4.35–5.09)	0.274, 0.112, 0.619
Fasting Glucose (mg/dL)	93.21 (90.02–96.40)	97.53 (96.27–98.79)	97.57 (96.53–98.60)	0.104, 0.463, **0.01**
REE (kcal/day)	1240 (1132–1358)	1217 (1176–1261)	1135 (1103–1167)	**0.002**, **0.001**, 0.242
Hours of sleep/night	8.35 (7.85–8.87)	8.83 (8.61–9.06)	8.95 (8.77–9.13)	0.056, 0.187, **0.044**

*rs1981429*	G/G	T/G	T/T	
n	56	119	77	
BMI	18.51 (17.82–19.23)	18.26 (17.80–18.73)	18.26 (17.68–18.86)	0.6283, 0.83 0.551
Weight (kg)	36.47 (35.13–37.86)	35.80 (34.92–36.71)	35.80 (34.69–36.94)	0.500, 0.764, 0.406
TFM (kg)	8.13 (7.15–9.24)	7.43 (6.81–8.10)	7.05 (6.32–7.87)	0.1124, 0.228, 0.166
LTM (kg)	25.22 (24.66–25.8)	25.45 (25.07–25.84)	26.01 (25.52–26.52)	**0.038, 0.035,** 0.223
IAAT (cm^2^)	30.78 (24.97–37.94)	27.85 (25.49–30.42)	28.85 (26.74–31.14)	0.944, 0.107, **0.049**
SI [×10^−4^ min^−1^/(µU/mL)]	4.80 (4.23–5.45)	5.00 (4.59–5.44)	4.87 (4.38–5.42)	0.918, 0.831, 0.676
Fasting Glucose (mg/dL)	97.85 (96.17–99.52)	97.44 (96.31–98.58)	96.69 (95.28–98.11)	0.298, 0.322, 0.492
REE (kcal/day)	1175 (1121–1231)	1192 (1156–1230)	1135 (1092–1180)	0.21, 0.322, 0.492
Hours of sleep/night	9.00 (8.70–9.30)	8.84 (8.64–9.04)	8.83 (8.59–9.07)	0.421, 0.688, 0.339

*Rs2267871*	A/A	T/A	T/T	
n	7	65	173	
BMI	19.26 (17.34–21.41)	18.17 (17.54–18.82)	18.39 (18.01–18.78)	0.963, 0.777, 0.364
Weight (kg)	37.95 (34.23–42.08)	35.47 (34.26–36.72)	36.16 (35.42–36.92)	0.843, 0.548, 0.317
TFM (kg)	7.72 (5.43–10.98)	7.11 (6.32–8.01)	7.70 (7.17–8.27)	0.454, 0.364, 0.915
LTM (kg)	27.18 (25.55–28.92)	25.42 (24.89–25.96)	25.55 (25.23–25.87)	0.503, 0.978, **0.045**
IAAT (cm^2^)	30.52 (21.68–42.97)	28.84 (26.06–31.93)	28.68 (26.78–30.71)	0.874, 0.963, 0.703
SI [×10^−4^ min^−1^/(µU/mL)]	5.09 (3.56–7.26)	4.31 (3.84–4.85)	5.04 (4.71–5.40)	0.086, **0.037**, 0.791
Fasting Glucose (mg/dL)	94.49 (89.71–99.28)	97.99 (96.47–99.50)	97.06 (96.12–98.00)	0.806, 0.49, 0.251
REE (kcal/day)	1311 (1159–1483)	1179 (1130–1231)	1162 (1132–1192)	0.128, 0.283, 0.067
Hours of sleep/night	7.91 (7.15–8.74)	9.17 (8.90–9.44)	8.81 (8.66–8.97)	0.461, 0.111, **0.021**

BMI: body mass index; TFM: total fat mass; LTM: lean tissue mass; IAAT: Intra-abdominal adipose tissue; SI: Insulin sensitivity index; REE: resting energy metabolism. Sex, age, Tanner stage, and African and European genetic admixture were used as covariates in all the analyses. Weight, TFM, and LTM were also adjusted for height. IATT, SI, fasting glucose, and REE were also adjusted for TFM. *P values represent the significance of the comparison among genotypes.

a , ^d^, and

r indicate *P* values calculated assuming additive, dominant, and recessive models, respectively. *P* values <0.05 are highlighted in bold case.

Nominal associations were also observed between alternative alleles at SNP rs1981429 and variation in lean tissue mass and intra-abdominal adipose tissue (Table 3), with individuals homozygous for the G allele having ∼3% less lean mass and 6% more intra-abdominal fat than those homozygous for the T allele.

Finally, we found a nominal association between alternative alleles at rs2267871 and variation in insulin sensitivity (Table 3). On average, children homozygous for the A allele were 15% more insulin sensitive than heterozygous children.

## Discussion

The syndecans are a family of cell-surface heparan sulphate proteoglycans that are expressed on the surface of all adherent cells [12]. In this study, we used quantitative complementation tests with two mutations of *dSdc* (*Sdc^10608^* and *Sdc^BG01305^*) to show that variation in this gene affects inter-individual variability in TAG storage between two strains of *D. melanogaster*. We then confirmed the effect of *dSdc* on TAG storage using a hypomorphic *dSdc* mutant (*Sdc^BG02774^*) and showed that flies homozygous for the mutation had significantly lower TAG storage than control flies under *ad libitum* feeding conditions. They also displayed significantly reduced metabolic rate and lower mitochondrial ADP-stimulated (state 3) respiration rate. Furthermore, female mutants had lower body weight, were leaner, had reduced glycogen levels, and were less fecund than controls. DILPs produced by IPCs in the brain of Drosophila have been shown to regulate body size, energy metabolism, and fecundity in several studies [29]–[31], [34], [45]. For example, Grönke *et al*. [34] reported that *dilp2-3,5* mutant flies had significantly reduced body weight, higher levels of lipids and glycogen, and were less fecund than controls. Another study found that flies homozygous for a deletion of *dilps1-5* not only were smaller and less fertile but also displayed reduced metabolic activity and TAG storage than controls [30]. Recent work has also suggested that activation of the insulin signaling in the fat body of adult flies leads to increased TAG storage via activation of shaggy, the fly ortholog of glycogen synthase kinase 3 [46]. In our study we observed that female *Sdc^BG02774^* flies had significantly lower expression of *dilp2*-*3*,*5* genes compared to controls. A significant reduction in *dilp2* expression was also observed in males. Thus, these data suggest that some of the effects associated with reduced dSdc function may be mediated by the reduced levels of DILPs. Further studies however will be required to determine not only if reduced expression of DILPs is causal in these effects, but also to understand how dSdc regulates the expression levels of *dilps* in Drosophila. We also observed that female mutants had significantly higher expression levels of *Akh*, which encodes a hormone involved in mobilization of glycogen reserve from the fat body [33], [47]. This finding could offer a plausible explanation for why female *Sdc^BG02774^* flies have lower levels of glycogen than controls.

Reduced insulin receptor/PI3-kinase/Akt signaling pathway results in nuclear accumulation of the transcription factor dFoxO and transcriptional activation of its targets, such as *Thor* [36]. However, despite reduced DILPs we observed a significant reduction in *Thor* expression in *Sdc^BG02774^* flies. This result is quite surprising considering that previous work reported that *Thor* transcript levels are up-regulated in *dilp2-3,5* mutants [34]. A possible explanation for our findings is that *dSdc* mutant flies have a defect in the mechanism(s) involved in dFoxO translocation and transcriptional regulation. This hypothesis is supported by the observation that, despite reduced *dilp2* transcript levels, male *dSdc* mutant flies did not show a compensatory increase in *dilp3* mRNA as a result of the reduced insulin signaling in the IPCs, an autocrine regulatory mechanism proposed by previous studies [34], [48]. These same studies have also shown that *dilp3* transcript in the IPCs was significantly reduced in dFoxO null flies [48] and suggested that *dilp3* acts as a positive regulator of *dilp2* and *dilp5* expression [34]. Hence, the hypothesis of a defect in dFoxo function may help explain the reduced levels of *dilp2-3,5* observed in female *Sdc^BG02774^* flies. In this context, another unanticipated finding in our study is that reduced expression of *dilps* did not lead to extended life span and increased starvation resistance as observed in several other studies [29], [34], [41]–[43]. On the contrary, homozygous *Sdc^BG02774^* flies showed significantly reduced survival. The mechanism behind this finding is unknown at present. However, it has been previously reported that *Thor* null flies had an impaired response to nutrient deprivation and shorter lifespan than controls [49], [50]. Based on these observations, it is tempting to speculate that the reduced levels of *Thor* transcript observed in the *Sdc^BG02774^* flies could be responsible for these phenotypes. Experiments are currently underway in our laboratory to test this hypothesis.

In mammals, PGC-1α and PGC-1β have been shown to play a pivotal role in the control of energy homeostasis. They regulate glucose and fat oxidation in muscle and fat tissue [37] as well as gluconeogenesis in liver [38] and glucose-regulated insulin secretion in pancreatic β cells [38]. In addition, they play an essential role in mitochondria biogenesis and function [39]. In Drosophila, a recent study suggested that Spargel, the fly homologue of PGC-1, is required for the stimulation of mitochondrial respiration, but not biogenesis [40]. *spargel* gene expression is induced by insulin signaling via a pathway parallel to dFOXO [40]. Consistent with these observations and the reduced levels of DILPs, we observed a significant decrease in the expression of *spargel* in *Sdc^BG0277^* ^4^ mutant flies compared to controls. *Sdc^BG0277^* ^4^ flies also showed a significant reduction in mitochondrial state-3 respiration rate, suggesting reducing mitochondrial oxidative phosphorylation capacity. In mammals, skeletal muscle metabolism influences whole-body metabolic rate [51], we thus speculate that *dSdc* might control Drosophila whole-body metabolic rate via the insulin signaling in skeletal muscle, which in turn regulates *spargel* expression and mitochondrial respiration.

In the present study we also found that *dSdc* mutant flies slept longer than controls. This increased sleep length is due largely to increases in the average duration of sleep bouts rather than their number (data not shown). Previous microarray analyses of fly heads revealed three genes with predicted functions in lipid metabolism that increased expression during sleep [52]. Furthermore, *P*-element insertions in metabolic pathway genes impacted sleep duration and bout number [53]. Like these recent studies, our results demonstrate a molecular link between energy stores and sleep. Though the nature of that link has yet to be elucidated, we hypothesize that the increased sleep in *Sdc^BG02774^* mutants in combination with reduced TAGs may be indicative of a strategy to conserve energy, an idea long postulated as a possible function of sleep [54]. This hypothesis is also supported by the significant reduction in metabolic rate of *dSdc* mutant flies.

One of the interesting findings of this component of the study was the sex-specific nature of the effects of *Sdc^BG02774^* on several phenotypes. Sex-specific effects on TAG storage were also observed in our initial complementation tests with different mutant alleles of *dSdc*. Such sex-specific allelic effects are commonly observed in Drosophila [55]–[57] and highlight the fact that genetic influences on phenotypes are sensitive to environmental conditions (in this case, differences in the internal physiological environment between sexes). As *dSdc* can act to modulate cell signaling, alternative alleles could be highly sensitive to different levels of signaling molecules that naturally occur between the sexes. Along these same lines, in the comparison of the control *CS* (*B*) flies, the baseline expression of *dSdc* was much higher in control females compared with the control males. Thus, we would expect to observe greater phenotypic effects of the mutation in females compared with males and this is what we observed in most traits. Understanding the mechanism underlying these sex specific allelic effects is an important goal for future study.

The results of the human study parallel the results of the experiments in flies. Overall, we observed that genetic variation in the human *SDC4* gene is associated with REE, sleep duration, and insulin dynamics in children. These results may shed light on physiological pathways underlying disease-related phenotypes in humans, perhaps by mechanisms that involve REE and/or sleep duration. Low REE has previously been associated with obesity- and diabetes-related traits in adults [58]–[60] and children [61]. Population differences in REE have been found, but the underlying reasons for these differences have not been elucidated. The role of genetics has been suggested in some studies [62]. Absolute levels of energy expenditure in humans are positively correlated with the amount of lean mass. However, individuals with higher levels of African genetic admixture tend to have lower levels of REE after adjustment for lean mass, suggesting a mechanism beyond simple energy balance [63]. This observation of lower REE is supported by research demonstrating a lower muscle oxidative capacity in AA women, suggesting that although they have higher levels of lean mass, the efficiency of fuel utilization that impacts energy expenditure is reduced [63]. In the current study, the *SDC4* genetic associations with REE and lean mass highlight the inter-relationship between these two traits. Previous studies reported that syndecan-4 is essential in skeletal muscle development and regeneration [64]. Moreover, syndecan-4 has been shown to play a direct role in the regulation of focal adhesion kinase phosphorylation [65], which in turn mediates the insulin sensitivity of skeletal muscle cells [66]. Thus, it is plausible that syndecan-4 is involved in energy regulation via an insulin-signaling mechanism that impacts overall body metabolism. This idea is corroborated by our Drosophila data.

Another point of potential interest is the relationship between the *SDC4* polymorphisms and sleep duration in early pubertal children. Previous reports have demonstrated that “sleep need” for this age group is approximately 9 hours per night [67], [68], and that a reduction in sleeping hours is a risk factor for obesity, due to its postulated effect on energy balance. Interestingly, the extent to which short sleep impacts obesity risk factors appears to be greater in children than adults [69]. Although a number of mechanisms have been proposed [67], some of which have a genetic basis [70], [71], no mechanism has been clearly identified to be in the causal pathway linking sleep and obesity. Given the strong similarity in results in Drosophila and humans, further exploration of the relationship between genes of the syndecan family, energy metabolism, and sleep is warranted.

## Materials and Methods

### Drosophila study

#### Fly stocks

The unrelated isogenic laboratory lines *2b* and *Ore* were used to establish the recombinant inbred lines in which QTL affecting TAG were previously mapped [10]. Mutant stocks were obtained from the Bloomington Drosophila Stock Center and from Trudy Mackay at North Carolina State University. The *Sdc^BG01305^* and *Sdc^BG02774^* lines were established by the Berkeley Drosophila Gene Disruption (BDGD) Project via *P*-element insertion into the second intron of the *dSdc* gene in the *w^1118^;Canton S* (*CS*) strain [26]. *Sdc^10608^* line is a *P*-element mutation that was established by the BDGD Project and maintained over a balancer (*Bal*) chromosome [72].

#### Experimental Design

To control for larval density, we allowed the parents of the experimental flies to mate for 3 hours to generate egg collections on apple juice/agar medium in laying plates. After 24 hours, we picked groups of 100 first-instar larvae from the surface of the medium and put into replicate vials. To minimize the influence of genetic variation in reproduction on energy metabolism, we performed all the metabolic assays on 3–5 day old virgin flies that were randomly collected from the replicate vials. We reared flies in vials containing 10 ml of standard cornmeal, agar, sugar, and yeast medium at a constant temperature of 25°C, 60–75% relative humidity, and a 12-hr light-dark cycle.

#### Quantitative complementation tests with *dSdc* mutations

We performed quantitative complementation tests by crossing virgin *Ore* and *2b* female flies to males of *dSdc* mutation stocks. These crosses produced four F1 genotypes: *M*/*2b*, *Bal or CS*/*2b*, *M*/Ore, and *Bal or CS*/*Ore*, where *M* denotes the *Sdc* mutation. We measured TAG storage in each genotypic class for each sex using the same experimental design described in [7]. We analyzed quantitative complementation test data for each sex separately, using the two-way mixed model factorial analysis of covariance (ANCOVA): *y*  =  µ + *L* + *G* + W + *L*×*G* + *E*, where *L* and *G* are the fixed cross-classified main effects of line (*Ore*, *2b*) and genotype (*M*, *Bal or CS*), W is the covariate body weight, and *E* is the within-vial variance. We inferred significant failure of the mutation to complement quantitative TAG phenotypes of *Ore* and *2b* alleles if the main effect of the *L*×*G* interaction term was significant, the contrast [*M*/*Ore* – *M*/*2b*]) was significant, and the contrast [*Bal or CS/Ore* – *Bal or CS*/*2b*] was not significant [73].

#### Body weight, TAG, protein, and glycogen measurements

We measured body weight, TAG and total protein levels using the protocol described in [7]. Briefly, groups of 10 single-sexed individuals were weighed to 0.01 mg accuracy with an analytical balance and homogenized in ice-cold KH_2_PO_4_ buffer. TAG content was measured for each homogenate spectrophotometrically using a commercially available kit (Sigma-Triglyceride Assay Kit) following the manufacturer's suggested protocol. Total protein levels were measured using a standard Lowry protein assay. Glycogen content was measured from the same homogenates using the protocol described in [74]. Briefly, aliquots of 1.67 µl of homogenate were added to 250 µl of a reagent containing 0.1 U/ml of amyloglucosidase. After 30-minute incubation period at 37°C, OD450 was measured. Concentration of glycogen was determined from glucose and glycogen standards run with each replicate. Each sample was assayed twice and the mean was used in the analysis. Analysis of variance (ANOVA) was used to determine statistical significance between mutant and control flies in body weight. Statistical differences in TAG, proteins, and glycogen were assessed by ANCOVA, with body weight used as covariate.

#### Female fecundity

We estimated female fecundity by standard procedures [75] using 20 females per genotype. Females were placed in egg laying chambers containing standard fly food and fecundity measured by counting the total number of eggs laid over a five day period. Statistical significance was determined by one-way ANOVA.

#### Metabolic rate

We measured metabolic rate as CO_2_ production using a flow-through respirometry system (Qubit System Research, Kingston, Ontario, Canada) and a modification of the method described in [76]. CO_2_ was measured for 10 minutes/chamber with a 30 second flush period between measurements. The amount of CO_2_ produced by each group of flies was calculated using C950 Data Acquisition software (Qubit System Research, Kingston, Ontario, Canada). Each group of flies was sampled at the same time of the day. The data analysis was performed using ANCOVA, with body weight used as covariate.

#### Sleep and waking activity

We maintained adult virgins at 30 flies to a single-sex vial to ensure that each line was exposed to identical levels of social [77] and had equal access to food. Sleep parameters for each fly were measured with the Drosophila Activity Monitoring System (Trikinetics, Waltham, MA), which counts the number of times a given fly crosses an infrared beam during a specified time interval. Here, we used one-minute intervals to record activity counts. Seven continuous days of sleep and activity were recorded for each experimental block. Sleep was defined as any period 5 minutes or longer without an activity count [78]. An in-house C++ program was used to calculate duration of sleep in minutes, numbers of sleep bouts, average sleep bout duration in minutes, and the number of activity counts per waking minute-waking activity. We used Wilcoxon T test to assess statistical significance between mutant and control in sleep and waking activity.

#### Survival assay

To examine the effects of the *dSdc* mutation on the lifespan of virgin flies, we measured survival using population cages. To obtain virgin offspring for each population cage, we standardized the parental density of each genotype (10 pairs) and allowed them to lay eggs for five days. Virgin offspring were collected over four successive days and added to a population cage at the end of this period. We set up three population cages of each sex and genotype (initial population sizes ranged from 183–295) and removed and counted dead flies every other day. We used Cox regression [79] as implemented by SAS (V9.1.3) to compare survival of mutants and controls. We analyzed survival for each sex separately, with replicate cage used as a covariate.

#### Starvation assay

We measured survival under starvation conditions by placing 10 flies per genotype/sex on 1.5% agarose medium and recording the number of flies alive at 8-h intervals until all were dead. Statistical significance was determined by one-way ANOVA.

#### Mitochondrial respiration rate

We placed live flies into 200 µl of ice-cold isolation buffer [250 mM sucrose, 5 mM Tris-HCl, 2 mM EGTA, 1% (w/v) bovine serum albumin (BSA), pH 7.4 at 4°C] supplemented with protease inhibitors (leupeptin 1 mg/ml, aprotinin 1 mg/ml and pepstatin 1 mg.ml) in a 1.5 ml Eppendorf tube. The samples were pounded gently 126 times over a 2 minute period, using a motorized micromortar, and filtered through a 5 µm nylon mesh. We then raised the volume to 400 µl by washing the nylon membrane with additional isolation buffer. After a cycle of low-speed centrifugation followed by a centrifugation of the filtered solution for 10 min at 3000 g, at 4°C, the pellet was re-suspended in 100 µl of isolation buffer. Protein concentrations in the mitochondrial fractions were determined using a Lowry assay. We performed mitochondrial respiration assays using freshly isolated mitochondria by measuring oxygen consumption in a two-chamber polarographic oxygen sensor (Oroboros oxygraph, OROBOROS® INSTRUMENTS, Innsbruck, Austria). We measured state 2, state 3 and state 4 respiration rates using NAD^+^-linked substrates (5 mM pyruvate + 5 mM proline) as implemented in [80]. Data were analyzed using the software *DatLab* Version 4.1.0.8. Statistical significance was determined by the two-tailed Student's *t* test.

#### Quantitative RT-PCR

We isolated total RNA using the TriPure RNA isolation kit (Roche). Isolated RNA was then used to make cDNA, using the First Strand Synthesis kit (Invitrogen). We performed RT-qPCR using a Syber Green Master mix and 50 ng total of cDNA per reaction and run in a Stratagene Mx3000P® qPCR machine. Statistical significance was determined by the two-tailed Student's *t* test.

### Human study

#### Subjects

A total of 252 children between the ages of 7 and 12 (52% male) were evaluated for the human association study. Subjects were participants from an ongoing study conducted at the University of Alabama at Birmingham. Race was determined by self-reported African-American, Caucasian or Hispanic ancestry in both parents and grandparents.

The study protocol was approved by the Institutional Review Board for human studies at the University of Alabama at Birmingham. A written informed consent was obtained from all study participants before enrolling in the study.

#### Phenotype measurements

Height and body weight in human subjects were measured in light indoor clothes and without shoes. Body composition (total fat mass and lean tissue mass) was measured by DXA using either a Lunar DPX-L densitometer (LUNAR Radiation Corp., Madison, WI) or a LUNAR Prodigy densitometer in the Department of Nutrition Sciences at UAB. Subjects were scanned in light clothing while lying flat on their backs with arms at their sides. Intra-abdominal adipose tissue was analyzed in the Department of Radiology by computed tomography scanning with a HiLight/Advantage Scanner (General Electric, Milwaukee).

Following an overnight fast, blood samples were obtained to establish the basal levels of glucose and insulin, and a frequently sampled intravenous glucose tolerance test (FSIGTT) was performed as described elsewhere [81]–[83]. Insulin sensitivity index (the increase in fractional glucose disappearance per unit of insulin increase) was estimated from the FSIGTT using minimal modeling [84].

REE was measured in the morning immediately after awakening during the overnight visit. A computerized, open-circuit, indirect calorimetry system with a ventilated canopy (Delta Trac II; Sensor Medics, Yorba Linda, CA) was used. While lying supine on a bed, the head of the subject was enclosed in a plexiglass canopy. Subjects were instructed not to sleep and remain quiet and still, breathing normally. One-minute average intervals of oxygen (VO_2_) uptake and VCO_2_ production were measured continuously for thirty minutes.

Sleep patterns were assessed by a questionnaire that was administered to the parents of the subjects. Parents were inquired about the amount of hours their child spent sleeping at night. We used hours sleeping at night as continuous phenotype.

#### Genotyping

We determined the genotypes of each *SDC4* polymorphism by Pyrosequencing technology [85] at the NORC Genetics Core at UAB. The genetic admixture estimates were obtained from the genotyping of ancestry informative markers across the human genome. Genotyping for the measures of genetic admixture was performed at Prevention Genetics (www.preventiongenetics.org). Approximately 100 ancestry informative markers (AIMs) were utilized for the study. Information regarding marker sequences, experimental details, and parental population allele frequencies has been submitted to dbSNP (http://www.ncbi.nlm.nih.gov/SNP/) under the handle PSU-ANTH [86], [87]. Individual West African, Amerindian, and European genetic admixture estimates were obtained by maximum likelihood approaches as reported in [88]. Briefly, the individual's genotypes at each AIM and the estimated allele frequencies of the AIMs in the three ancestral parental populations were used to estimate the admixture proportion that corresponds to the maximum combined probability across all loci for West African, Amerindian and European proportions. These estimates were used as covariates in all statistical models to control for population stratification.

#### Statistical analyses

We assessed Hardy-Weinberg equilibrium, allelic frequencies, and D' linkage disequilibrium coefficients using Haploview v3.2 [89]. We performed genotype frequency comparisons between EA, AA, and HA samples by ANOVA. To test the effect of each genotyped SNP on trait variation, we performed genotypic associations for dominant, additive, and recessive models using linear regression analysis. Dummy variables were assigned to code the three genotypes in each model. In the additive model, we used 0, 1 and 2 to code for individuals homozygous for the major allele, heterozygous, and homozygous for the minor allele, respectively. In the dominant and recessive models, we used 0 to code for individuals homozygous for the major and minor alleles, respectively, and 1 to code for individuals carrying at least one copy of the other allele. For all regression models, studentized residuals were evaluated for normality and logarithmic transformations of the dependent variable was performed to improve normality. When normality of the residuals was not obtained after transformations, the observations that were above and below three standard deviations were removed from the analyses. Analyses were performed using PLINK [90] and SAS 9.1 software (SAS Institute, Cary, NC).
